# Case Report: A case of third-degree atrioventricular block associated with primary cardiac lymphoma

**DOI:** 10.3389/fcvm.2024.1356134

**Published:** 2024-02-28

**Authors:** Jianping Liu, Yong Zheng, Weishan Zhang, Juan Xia, Yongheng Zhang, Long Tang

**Affiliations:** ^1^ Department of Cardiovascular Surgery, Suining Central Hospital, Suining, Sichuan, China; ^2^ Department of Pathology, Suining Central Hospital, Suining, Sichuan, China; ^3^ Department of Hospital-Acquired Infection Control, Suining Central Hospital, Suining, Sichuan, China

**Keywords:** cardiac lymphoma, atrioventricular block, diffuse large B-cell lymphoma, case report, diagnosis, treatment

## Abstract

**Background:**

Primary cardiac lymphoma is an extremely rare malignant lymphoma, with clinical manifestations related to its location. We reported the diagnosis and treatment of primary cardiac lymphoma in a patient presented with atrioventricular block.

**Case presentation:**

A 64 year-old man was admitted to our hospital because of symptoms of a tired heart and shortness of breath. The initial electrocardiogram revealed a third-degree atrioventricular block. Computed tomography scan showed an irregularly shaped right heart, irregular clusters, and relatively weakly enhanced areas in the right auricle, atrium, and ventricle. The local boundary between the lesion, pericardium, and left atrium was unclear, and the ventricular septum was irregular and thickened. Multiple irregular gray neoplasms with less smooth surfaces were observed, with a maximum diameter of approximately 7 cm. Pathological findings confirmed a non-germinal center B cell subtype of diffuse large B-cell lymphoma. After surgical resection of the tumor and implantation of a permanent pacemaker, the symptoms of the patient were significantly improved, allowing subsequent chemotherapy.

**Conclusion:**

Surgical resection and placement of a permanent pacemaker were effective treatments for a patient with primary cardiac lymphoma presented with atrioventricular block.

## 1 Introduction

Primary cardiac tumors are particularly rare, with a prevalence ranging from 0.001% to 0.03%, with 1.3% of primary cardiac tumors and 0.5% of extranodal lymphomas being primary cardiac lymphomas (1). Primary cardiac lymphoma, which always involves the right atrium and ventricle, is three times more common in women than men, with 64 years being the median age for disease diagnosis (2). Primary cardiac lymphoma is defined as follows: (1) the heart or pericardium is affected by malignant lymphoma as demonstrated by autopsy (3); and (2) tumor tissue consisting of cardiac or pericardial lymphoma or lymphoma infiltration in the myocardium causing cardiac symptoms at initial diagnosis (4). Primary cardiac lymphomas are largely reported as isolated cases owing to their rarity and difficulty in their diagnosis. Thus, owing to late diagnosis, the prognosis of primary cardiac lymphoma is poor, with the median survival after diagnosis being 7 months (5).

The main clinical manifestations of primary cardiac lymphoma are cardiac symptoms induced by lymphoma infiltration of the myocardium. Other signs may be observed, including mediastinal lymph node enlargement, pleural exudation, and pulmonary embolism (6). Primary cardiac lymphoma is easily misdiagnosed in patients presenting with atrioventricular block only (7). We reports a case of third-degree atrioventricular block secondary to primary cardiac lymphoma that provides theoretical and empirical evidence for the diagnosis and treatment of this disease.

## 2 Case description

**Figure 1:**
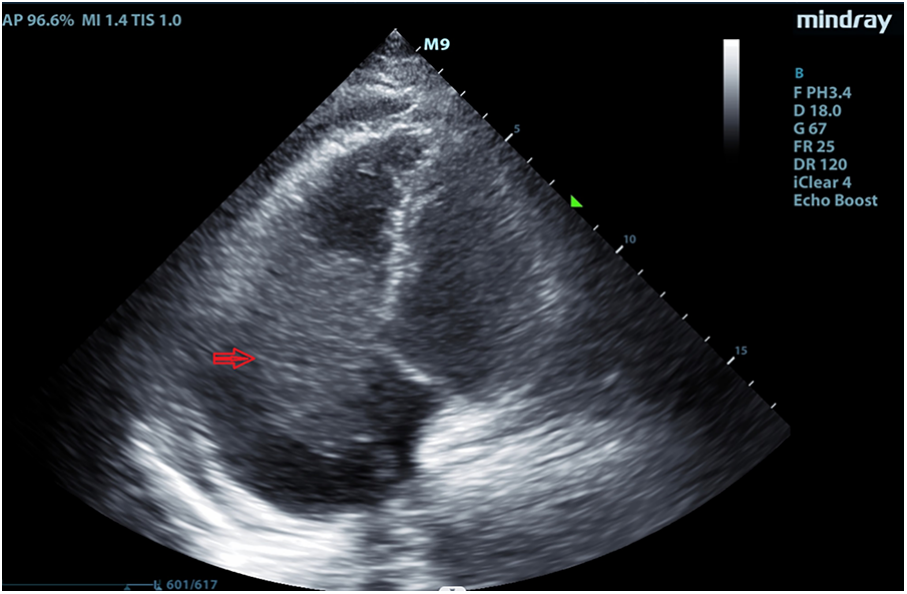
Echocardiography revealed a hypoechoic mass extending from the right atrium to the right ventricle in September 2, 2022.

**Figure 2:**
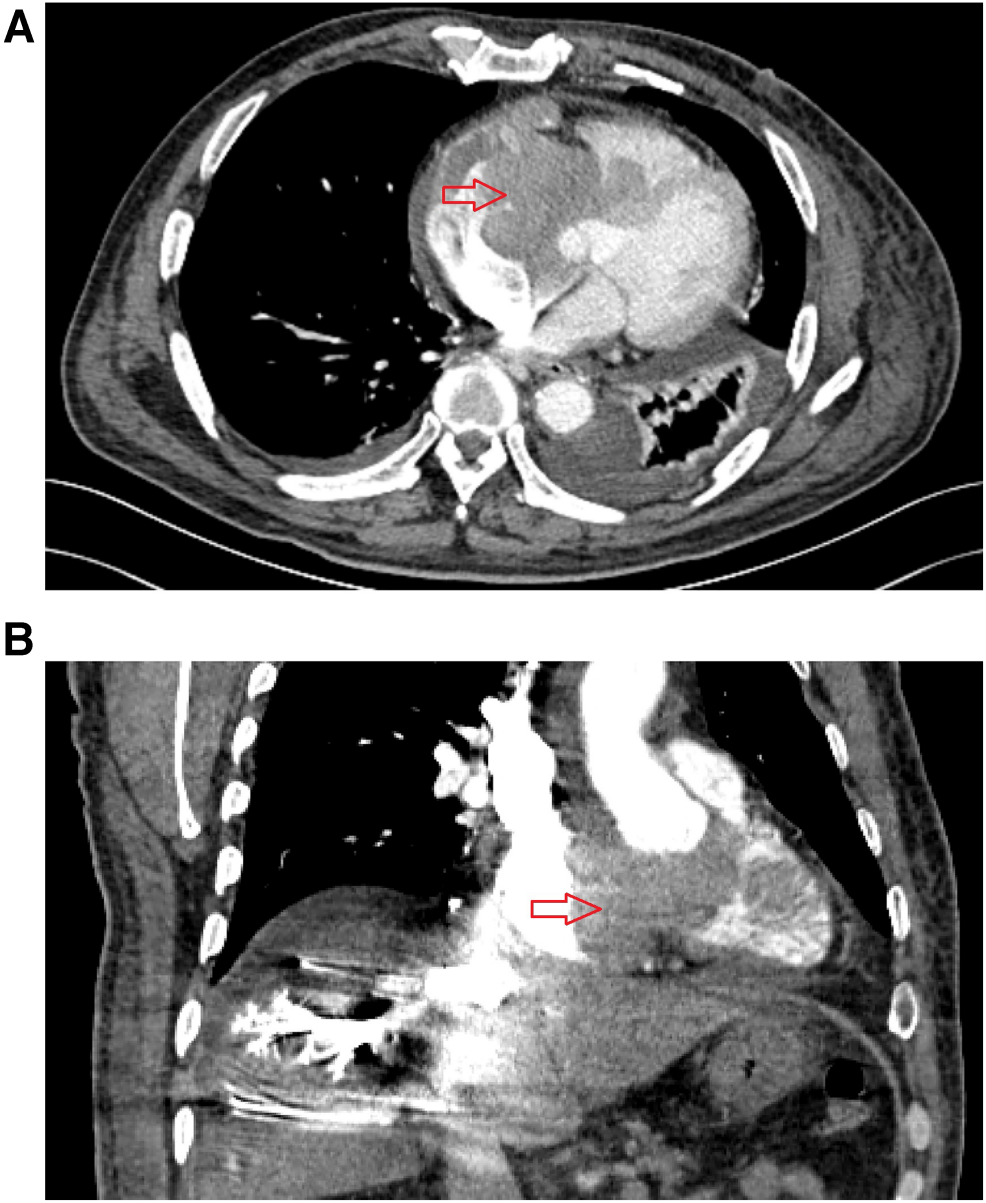
Preoperative enhanced computed tomography results for patient in September 2, 2022. (**A**) The cross-sectional image shows a mass in the right atrium, with irregular patchy enhancement in the right atrial appendage, right atrium, and right ventricular region, with a CT value of approximately 89 Hounsfield units. The interventricular septum is irregularly thickened. (**B**) Coronal view shows a mass in the right atrium, uneven opacification in some areas of the superior vena cava, delayed opacification of the inferior vena cava and veins in the right lobe of the liver.

A 64 year-old man was admitted to our hospital on September 2, 2022, owing to symptoms of a tachycardia and shortness of breath. Physical examination at admission revealed the following: body temperature: 36.5°C, heart rate: 51 times/min, pulse: 51 beats/min; blood pressure: 102/68 mmHg, poor spirit, edema of the trunk, yellow sclera, slight cyanosis of the lip, no filling of the jugular vein, coarse breath sounds in both lungs, no enlargement of the heart boundary, arrhythmia, soft abdomen, and severe pitting edema of the lower limbs. Moreover, laboratory tests revealed the following: cardiac troponin I (cTNI) < 0.02 ng/ml (normal range: <0.53 ng/ml), creatinine-kinase-MB: 5.49 ng/ml (normal range: <5.0 ng/ml), myohemoglobin: 216.82 ng/ml (normal range: <110 ng/ml), D-dimer: 1214.27 ng/ml (normal range: <200 ng/ml), and B-type natriuretic peptide: 2460.72 pg/ml (normal range: <100 pg/ml). Electrocardiography indicated sinus rhythm, third-degree atrioventricular block, full-lead low voltage, QT interval prolongation, and borderline escape rhythm. Echocardiography revealed a hypoechoic mass extending from the right atrium to the right ventricle and obstructing the forward flow of the tricuspid valve. The size of the mass was approximately 66 × 40 mm (Figure 1). Computed tomography revealed the following: (1) irregular shape of the right heart, with the enhanced right auricle, right atrium, and ventricle exhibiting irregular clusters and relatively weakly enhanced areas. The CT value after enhancement was approximately 70–90 HU, while the larger cross-section was approximately 6.2 × 6.9 cm. The local boundaries between the lesion, pericardium, and left atrium were unclear, while the ventricular septum was irregular and thickened; (2) the filling of the superior vena cava and left and right brachiocephalic veins was not uniform; (3) the pulmonary artery and its branches in the upper, middle, and lower lobes of the right lung were poorly developed, while the local lumen was suspected to be uneven, exhibiting a few patchy and slightly low-density shadows. A suspicious low density shadow was observed at the edge of the upper and lower pulmonary arteries of the left lung; (4) the two lungs were dispersed with flaky shadows of increased density, the volume of the lower lobe of the left lung was reduced, a dense shadow was present in some lung tissues, and the enhancement was visible; (5) the bronchial segment of the left inferior lobe of the lung was slightly narrowed, while part of the bronchus of the left inferior lobe was unclear; (6) pericardial effusion, bilateral pleural effusion, left interlobar fissure, and adjacent lung hypoinflation; (7) the thoracic section of the esophageal wall was slightly thickened, approximately 0.4 cm; (8) swelling of left axilla, chest wall, and back soft tissue; (9) abdominal fluid, the local adipose space in the abdominal area was seen as lamellar and slightly high-density shadows; (10) multiple small veins in inferior vena cava and right lobe of liver were developed in advance; and (11) the adipose space in the abdominal cavity was blurred, with multiple pieces being increasingly flocculent, while the fascia around both kidneys was thickened (Figure 2).

September 3, 2022, hematological tests revealed the following: reduced lymphocyte ratio (8.1%, normal range: 20%–50%), platelet count (88 × 10^9^, normal range: 100–300 × 10^9^), prothrombin time activity (36%, normal range: 70%–130%), and level of plasma fibrinogen (1.7 g/L, normal range: 2–4 g/L), whereas elevated neutrophil ratio (76.3%, normal range: 40%–75%), level of high-sensitivity C-reactive protein (40.25 mg/L, normal range: 0–10 mg/L), prothrombin time (23.1 s, normal range: 9.6–12.8 s), international normalized ratio (2.05, normal range: 0.88–1.15), activated partial thromboplastin time (46 s, normal range: 24.8–33.8 s), and levels of D-dimer (8.52 μg/ml, normal range: <200 ng/ml), fibrinogen degradation product (25.5 μg/ml, normal range: 0–5 μg/ml), cTNI (74.8 pg/ml), myohemoglobin (476.54 ng/ml), N-terminal brain natriuretic peptide (700.7 pg/ml, normal range: <100 pg/ml), alanine transaminase (ALT) (1,202 U/L, normal range: 9–50 U/L), aspartate transaminase (AST) (806 U/L, normal range: 15–40 U/L), total bilirubin (72.1 μmol/L, normal range: <26 μmol/L), direct bilirubin (40.8 μmol/L, normal range: <4 μmol/L), indirect bilirubin (31.3 μmol/L, normal range: <22 μmol/L), urea (27.97 mmol/L, normal range: 3.6–9.5 mmol/L), creatinine (203 μmol/L, normal range: 57–111 μmol/L), and uric acid (1,265 *Μ*mol/L, normal range: 208–428 μmol/L). On September 4, 2022, the patient underwent preoperative preparation, and the right atrial and right ventricular masses were removed using tracheal intubation cardiopulmonary bypass. Pericardial mediastinal drainage was performed and a temporary cardiac pacemaker was implanted during surgery. Intraoperative findings included the following: (1) multiple irregular gray neoplasms in the right atrium, which was characterized by less smooth surfaces and a maximum diameter of approximately 7 cm. The base was located in the atrial septum, which had grown compact with the tricuspid valve without gaps. An increased number of mural thrombi were present around the tumor body of the right atrium, with some thrombi being embedded in the dressing muscle of the right atrium; (2) several new organisms with a diameter of approximately 3 cm were observed in the right ventricular chamber, which straddled the tricuspid valve opening, resulting in severe blockage of the tricuspid valve; (3) the tumor profile was yellow and fish-like; (4) some tumors were not densely adhered to the tricuspid septum, while the tricuspid ring was slightly enlarged, and the water injection test showed mild regurgitation. Postoperative pathological examination revealed a bunch of grayish white tissue with a total volume of 7.5 × 6.5 × 3 cm. Focal hemorrhage and necrosis were observed in this section. Microscopically, the lymphocytic phenotype was diffuse and scattered among histiocytes, small lymphocytes, and nuclear fragments. Immunohistochemical examination revealed the following: medium large heterogeneous lymphocyte CD20 (+), CD19 (+), CD22 (+), CD10 (−), BCL-6 (+), MUM-1 (+), CD30 (−), BCL-2 (+, 90%), C-MYC (+, 50%), Ki-67(+, 90%), CD3 (−), CD5 (−), CyclinD1 (−), TDT (−), p53 (+, 70%), and PCK (−). Fluorescence *in situ* hybridization analysis showed no separation rearrangement of BCL-6, BCL-2, and MYC. Therefore, the patient was diagnosed as having the non-germinal center B cell (non-GCB) subtype of diffuse large B-cell lymphoma. BCL-2, C-MYC double expression. Bone marrow biopsy and flow cytometry revealed no lymphocytic infiltration. On September 5, 2022, the patient presented elevated levels of ALT (204 U/L), AST (297 U/L), total bilirubin (100 μmol/L), direct bilirubin (35 μmol/L), indirect bilirubin (65 μmol/L), urea (27.88 mmol/L), creatinine (280 μmol/L), uric acid (1,045 μmol/L), as well as increased prothrombin time (17.8 s), international normalized ratio (1.46), and activated partial thromboplastin time (50.9 s), whereas prothrombin time activity (55%), and the level of plasma fibrinogen (1.93 g/L) were significantly reduced. A permanent pacemaker was placed after the patient stabilized.

**Figure 3:**
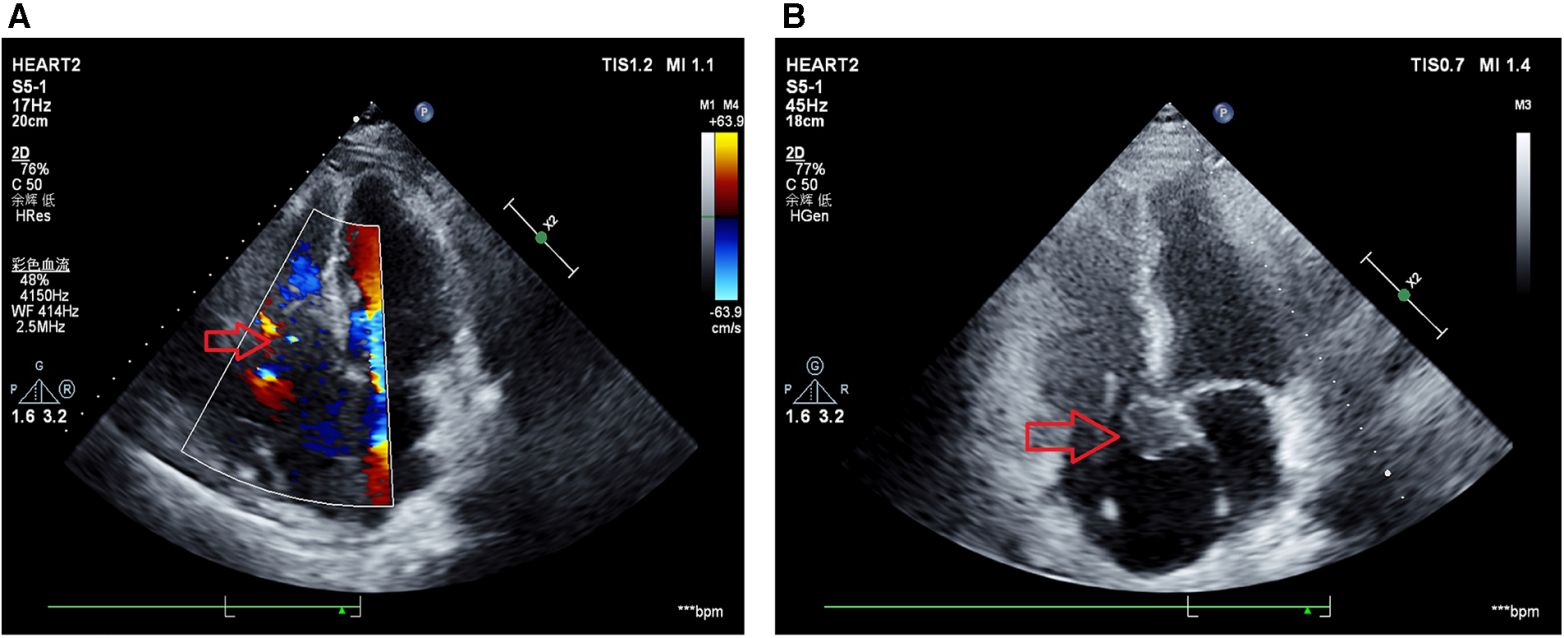
Echocardiography results after surgical resection. (**A**) Echocardiogram results at October 19, 2022 indicated a hypoechoic mass with irregular shape measuring approximately 85 mm × 54 mm is observed from the right atrium to the right ventricle. The boundary between the mass and the tricuspid valve is unclear, and the mass infiltrates the interatrial septum, suggesting tumor recurrence (with an increased size compared to pre-surgery) and severe tricuspid valve stenosis. (**B**) Echocardiogram results at November 1, 2022 indicated irregularly shaped solid mass in the right atrium, measuring 25 mm × 21 mm, adhering to the septal leaflet and inferior interatrial septum, with enlargement of the left and right atria, dilation of the aortic sinus, and severe tricuspid regurgitation.

On October 19, 2022, echocardiography revealed an enlarged left atrium and a hypoechoic mass extending from the right atrium to the right ventricle with an irregular shape and a size of approximately 85 × 54 mm was (Figure 3A). The boundary between the mass and tricuspid septum was unclear, while the atrial septum was infiltrated by the mass, suggesting tumor recurrence and severe tricuspid stenosis. The patient was administered an rituximab, cyclophosphamide, doxorubicin, vincristine, and prednisone (R-CHOP) regimen. On November 1, 2022, echocardiography revealed the irregular shape of the right atrium, a solid mass measuring 25 × 21 mm, adhesion to the root of the tricuspid septum and lower atrial septum, enlargement of the left and right atria, widening of the aortic sinus, severe tricuspid regurgitation, and arrhythmia, with an ejection fraction of 71% (Figure 3B). Chest computed tomography showed exudation of the mediastinum and pericardium, slightly increased levels of blood and fluid, pneumonia, bilateral pleural effusion with atelectasis of the adjacent lung tissue, slightly narrowed left dorsal segment bronchus of the lower lobe of the lung due to compression, and aortic and coronary area calcification. Next-generation sequencing of the mass indicated a class I variation of *CD79B* (+), C.587A > C, which suggesting The adenine (A) at the 587th base position of the CD79B gene is replaced by cytosine (C). After three months post-surgery, the patient died due to severe lung infection following chemotherapy.

## 3 Discussion

Primary cardiac lymphoma, of which diffuse B-cell lymphoma is the main pathological type, is extremely rare. The disease often involves the right atrium, right ventricle, atrial septum, and inferior vena cava. We reported a patient with highly aggressive CD20 (+) B-cell lymphoma, which invaded the right atrium, right ventricle, and atrial septum and involved the tricuspid septum, resulting in severe tricuspid stenosis. The clinical manifestations of primary cardiac lymphoma lack specificity and are closely related to tumor size, location, and complications. Right heart tumors can cause venous congestion, and their enlargement aggravates the disease. Tumors can invade the atrioventricular valve and cause stenosis or incomplete closure of the valve. The main clinical symptoms include congestive heart failure (31%–78%), chest pain (19%–46%), and arrhythmia (8%–56%) (8, 9). Other less common symptoms include the superior vena cava syndrome (10–12), odynophagia (6), and embolic phenomena (13). In this paper, we noted that the patient presented with dyspnea, edema, multiple serous effusions, hepatic and renal dysfunction, coagulation dysfunction, sinus rhythm, third-degree atrioventricular block, full-lead low voltage, QT interval prolongation, and a borderline escape rhythm.

Numerous studies have reported cases of primary cardiac lymphoma accompanied by atrioventricular block (Table 1) (14–25). The age of patients included in these studies ranged from 36.0 to 80.0 years, with 58.3% of them being men. Of the 12 included patients, 2 had primary cardiac T-cell lymphoma and most had complete atrioventricular block. All patients underwent surgical resection, pacemaker implantation, and chemotherapy, resulting in the complete remission or reduced mass size of tumors and a better prognosis. The primary treatment for cardiac lymphoma was chemotherapy; however, some patients presented with extensive myocardial involvement, which could cause sudden cardiac arrest (26, 27). Thus, surgical resection was performed first in patients at high risk of sudden cardiac events due to cardiac involvement. An alternative method for treating atrioventricular block was surgical epicardial lead implantation, particularly in patients presenting with broad cardiac involvement. Complete atrioventricular block was always caused by infiltration of the conduction system by primary cardiac lymphoma, and pacemaker lead implantation was always performed owing to bradycardia-induced heart failure (27).

**Table 1:** The summary results for primary cardiac lymphoma with atrioventricular block.

Study	Gender	Age (years)	Disease status	Clinical symptoms	Imaging diagnosis	Treatments	Prognosis
Frikha et al. (14)	Male	64.0	Non-Hodgkin large B-cell lymphoma	Cardiac tamponade and paroxysmal third-degree atrioventricular block	Echocardiography, computed tomography, coronary angiography	Surgical resection and R-CHOP	The size of the mass was reduced
Crisel et al. (15)	Male	55.0	Diffuse large B-cell lymphoma	Complete atrioventricular block	Echocardiography, cardiac magnetic resonance imaging	Pacemaker implantation and R-EPOCH	No identifiable tumor
Chen et al. (16)	Male	36.0	Primary diffuse cardiac large B-cell lymphoma	Atrioventricular block and paroxysmal ventricular tachycardia	Echocardiography, computed tomography	COP, CHOP, and R-CHOP	Adequate global systolic and diastolic functions, with significant reduction of myocardial thickness and resolution of pericardial effusion
Wang et al. (17)	Female	70.0	Primary cardiac T-cell lymphoma	Complete atrioventricular block and torsades de pointes	Echocardiography, computed tomography	CHOP	Complete remission
Sekar et al. (18)	Male	76.0	High-grade non-Hodgkin's lymphoma of B-cell type	Acute coronary syndrome and atrioventricular block	Echocardiography, computed tomography	R-GCVP	Tumor recurrence
Jang et al. (19)	Male	56.0	Diffuse large B-cell lymphoma	Atrioventricular block	Echocardiography, cardiac magnetic resonance imaging, positron emission tomography-computed tomography	Surgical resection, pacemaker implantation, R-CHOP	Complete remission
Usry et al. (20)	Female	80.0	C-MYC positive, EBV-negative, Burkitt lymphoma	Symptomatic complete heart block	Echocardiography, cardiac magnetic resonance imaging, coronary angiography	Pacemaker implantation and R-CHOP	The size of the mass was reduced
Al Mawed et al. (21)	Female	73.0	Diffuse large B-cell non-Hodgkin lymphoma of non-germ-cell type	Atrial flutter and atrioventricular block	Echocardiography, cardiac magnetic resonance imaging, positron emission tomography-computed tomography	Pacemaker implantation, R-mini-CHOP and R-CHOP	Complete remission
Hu et al. (22)	Male	70.0	Non-germinal centre diffuse large B-cell lymphoma	Complete atrioventricular block	Echocardiography, computed tomography, positron emission tomography-computed tomography	R-EPOCH	The size of the mass was reduced
Chen et al. (23)	Female	47.0	Primary cardiac T-cell lymphoma	Complete atrioventricular block	Echocardiography, cardiac magnetic resonance imaging, computed tomography, positron emission tomography-computed tomography	MTX-CHOP	Complete remission
Shigeno et al. (24)	Female	55.0	Diffuse large B-cell lymphoma	Complete atrioventricular block	Echocardiography, computed tomography	Surgical resection, epicardial lead implantation, R-CHOP	Complete remission
Mao et al. (25)	Male	64.0	Primary diffuse cardiac large B-cell lymphoma	Complete atrioventricular block	Echocardiography, computed tomography, positron emission tomography-computed tomography	Pacemaker implantation, R-COP and R-CDOP	The size of the mass was reduced

Accurate diagnosis at an early stage is particularly important for patients with primary cardiac lymphoma (28). Imaging examinations could be considered useful tools for detecting masses in the heart; however, imaging examinations do not provide a definitive diagnosis. However, echocardiography can provide information regarding the location, size, and activity of the tumor, tumor involvement in the atrioventricular valve, and cardiac function. Once echocardiography suggests a malignant tumor in the heart, computed tomography and cardiac magnetic resonance imaging can be performed to observe tumor infiltration and the relationship with surrounding tissues, providing information for surgical planning. Furthermore, positron emission tomography-computed tomography can comprehensively determine the presence of distant metastasis and the presence of other extracardiac tumors. These diagnostic tools can provide further information guiding personalized treatments. Of note, endomyocardial biopsy is considered a valuable tool for diagnosing intracardiac masses and arrhythmogenic cardiomyopathies (23). In our paper, the patient underwent echocardiography and computed tomography to detect the tumor location, while an endomyocardial biopsy was performed to assess the primary cardiac lymphoma.

## 4 Conclusions

This paper described the clinical course of a patient with a non-GCB subtype of diffuse large B-cell lymphoma diagnosed via endomyocardial biopsy. Echocardiography and computed tomography were used as imaging diagnostic tools, and the patient underwent surgical resection of the right atrial and right ventricular masses through tracheal intubation cardiopulmonary bypass. Furthermore, a permanent pacemaker was placed after the patient stabilized, followed by administration of R-CHOP as the chemotherapy regimen. After chemotherapy, the size of the tumor was reduced, indicating remission.

## Data Availability

The original contributions presented in the study are included in the article/Supplementary Material, further inquiries can be directed to the corresponding author.
